# MicroRNA expression analysis in the liver of high fat diet-induced obese mice

**DOI:** 10.1016/j.dib.2016.11.081

**Published:** 2016-12-01

**Authors:** Won-Mo Yang, Kyung-Ho Min, Wan Lee

**Affiliations:** a Department of Biochemistry, Dongguk University College of Medicine, Gyeongju 780-714, South Korea; b Endocrine Channelopathy, Channelopathy Research Center, Dongguk University College of Medicine, Goyang 410-773, South Korea

**Keywords:** MicroRNAs, High fat diet, Saturated fatty acids, Obesity, Liver, Mice

## Abstract

A previous study indicated a causal link between certain miRNAs induced by obesity and the development of hepatic insulin resistance and type 2 diabetes. Here we provide accompanying data collected using Affymetrix GeneChip miRNAs microarrays to identify the changes in miRNAs expression in the liver of mice fed a high fat diet (HFD). Differentially expressed microRNA analyses in the liver of the HFD-fed mice revealed a range of upregulated (>1.5-fold) or downregulated (<0.5-fold) miRNAs. Among those upregulated miRNAs, *in silico* target analysis, such as TargetScan, PicTar, and miRWalk, identified miRNAs with the putative binding sites on the 3’UTRs of *INSR* and/or *IRS-1*. Interpretation of the data and further extensive insights into the implication of miRNAs, particularly miR-15b, in hepatic insulin resistance can be found in "Obesity-induced miR-15b is linked causally to the development of insulin resistance through the repression of the insulin receptor in hepatocytes." (W.M. Yang, H.J. Jeong, S.W. Park, W. Lee, 2015)[1].

**Specifications Table**

**Table:** 

Subject area	*Biology, Biochemistry*
More specific subject area	*Obesity, Metabolism, MicroRNA*
Type of data	*Tables and Excel files*
How data was acquired	*Affymetrix GeneChip microarray analyses of miRNAs*
Data format	*Analyzed*
Experimental factors	*Diet-induced obesity, RNA Isolation, Affymetrix Genechip miRNA microarray, In silico analysis of miRNAs*
Experimental features	*Differentially expressed miRNAs of the liver of mice fed with either a NFD or a HFD were analyzed using Affymetrix GeneChip miRNA microarray.*
Data source location	*Dongguk University School of Medicine, Gyeongju 780-714, Korea*
Data accessibility	*The data are available with this article*

**Value of the data**

• The data can allow a prediction of the biological significance of miRNAs associated with the pathogenesis of obesity, insulin resistance and type 2 diabetes.
• The data can be compared with miRNA analysis from other cell or tissue types with obesity.
• Differentially expressed miRNAs in this dataset could be applied to further study the changes in the cellular phenotype by high fat diet-induced obesity and metabolic diseases.
• The results support a previous study [1] and the use of transcriptomic technologies in non-model organisms.

## 1 Data

Diets rich in saturated fatty acids (SFA) can exacerbate obesity [2] and increase the risk of insulin resistance. This condition is characterized by an inadequate response of the insulin-sensitive tissues to insulin, leading to type 2 diabetes and metabolic syndrome [3]. Obesity modulates aberrantly the expression of certain miRNAs targeting the mRNAs of the insulin signaling molecules, and participates actively in the pathogenesis of insulin resistance [4], [5]. A previous study reported that a high fat diet (HFD) induces miR-15b in the liver of mice, which suppresses the expression of hepatic INSR, but not IRS-1, by targeting *INSR* 3’UTR directly [1]. Therefore, certain types of miRNA induced by obesity can be linked causally to the development of hepatic insulin resistance, which may in turn lead to type 2 diabetes. This study provides accompanying data collected using Affymetrix GeneChip microarrays to identify the changes in miRNA expression in the liver of mice fed with a HFD for 14 weeks. Differentially expressed miRNA analyses in the liver of HFD-fed mice showed that a range of miRNAs were upregulated more than 1.5-fold (Supplement File. 1) or downregulated less than 0.5-fold (Supplement File. 2). Among those differentially expressed miRNAs, the upregulated miRNAs may be involved in the reduction of INSR and IRS-1 levels observed in the liver of HFD-fed mice. Therefore, this study next examined whether the 3’UTRs of *INSR* and *IRS-1* possess direct binding sites for the upregulated miRNAs. *In silico* target analysis using TargetScan, PicTar, and miRWalk showed that a range of certain miRNAs have putative binding sites for the 3’UTRs of *INSR* (Table 1) and/or *IRS-1* (Table 2). An interpretation of the data and further extensive insights into the implication of miRNAs, particularly miR-15b, in hepatic insulin resistance can be found elsewhere [1].

## 2 Experimental design, materials and methods

### 2.1 Animals and high fat diet (HFD)-induced obesity

All The Animal Use and Care Committee at Dongguk University approved all experimental procedures involving mice. The C57BL/6N male mice were purchased from OrientBio (Seongnam, Gyeonggi, Korea). They were maintained in a temperature (20–22 °C) and humidity (55±10%) controlled facility with a 12:12 h light–dark cycle and given access to food and water *ad libitum*. At 6 weeks of age, the mice were fed either a normal fat diet (NFD, 12.4% calories from fat; Purina, Wilkes-Barre, PA, USA) or a HFD (60% calories from fat; Dyets Inc., Bethlehem, PA, USA) for 14 weeks. During the experimental period, the body weights of the mice were recorded weekly. At the end of the experiment, mice were fasted for 12 h and sacrificed by a cervical dislocation. The liver was removed rapidly, washed with cold PBS, and subjected to RNA extraction.

### 2.2 RNA extraction and quality check

The total RNA from the liver of mice was extracted using a miRNeasy Mini Kit (Qiagen) according to the manufacturer׳s instructions. The purity and integrity of the RNA extracted were assessed using a ND-1000 Spectrophotometer (NanoDrop) and Agilent 2100 Bioanalyzer (Agilent Technologies). Equal amounts of RNA from five mice were combined and used for the microarray.

### 2.3 miRNA arrays analysis

The total RNA from the liver of the mice described above was prepared and subjected to the Affymetrix Genechip miRNA 4.0 array (Affymetrix) process according to the Affymetrix technical instructions. Briefly, 600ng of RNA was labeled with a FlashTag™ Biotin RNA Labeling Kit (Genisphere, Hatfield, PA, USA). The labeled RNA was quantified, fractionated, and hybridized to the miRNA microarray provided by the manufacture. The labeled RNA was heated to 99 °C for 5 min and incubated to 45 °C for 5 min. RNA-array hybridization was conducted with agitation at 60 rpm for 16 h at 48 °C on an Affymetrix^®^ 450 Fluidics Station. The chips were stained using a Genechip Fluidics Station 450 (Affymetrix), and scanned with an Affymetrix GCS 3000 scanner (Affymetrix). All signals were normalized using the quantile method after a log_2_ transformation to make them comparable across the microarrays.

### 2.4 Data extraction and in silico analysis

Differentially expressed miRNAs were analyzed automatically using Affymetrix^®^ GeneChip™ Command Console software according to the Affymetrix data extraction protocol and identified by calculating the fold change of either a NFD-fed mice versus a HFD-fed mice. The differentially expressed miRNAs putatively targeting *INSR* 3’UTR and *IRS-1* 3’UTR were screened computationally using publicly available algorithms (TargetScan: www.targetscan.org, Pictar: pictar.mdc-berlin.de, and miRWalk: www.umm.uni-heidelberg.de).

## Figures and Tables

**Table 1:** miRNAs putatively targeting *INSR*.

**miRNAs**	**Fold change**	**Accession**	**Sequence**
miR-15b-5p	1.62	MIMAT0000124	UAGCAGCACAUCAUGGUUUACA
miR-28a-5p	1.58	MIMAT0000653	AAGGAGCUCACAGUCUAUUGAG
miR-28a-3p	1.86	MIMAT0004661	CACUAGAUUGUGAGCUGCUGGA
miR-132-3p	2.15	MIMAT0000144	UAACAGUCUACAGCCAUGGUCG
miR-140-3p	1.73	MIMAT0000152	UACCACAGGGUAGAACCACGG
miR-149-5p	16.27	MIMAT0000159	UCUGGCUCCGUGUCUUCACUCCC
miR-151-3p	1.93	MIMAT0000161	CUAGACUGAGGCUCCUUGAGG
miR-181a-5p	2.21	MIMAT0000210	AACAUUCAACGCUGUCGGUGAGU
miR-183-5p	1.51	MIMAT0000212	UAUGGCACUGGUAGAAUUCACU
miR-193a-5p	1.77	MIMAT0004544	UGGGUCUUUGCGGGCAAGAUGA
miR-212-3p	3.23	MIMAT0000659	UAACAGUCUCCAGUCACGGCCA
miR-292b-5p	2.83	MIMAT0029864	ACUCAAAACCUGGCGGCACUUUU
miR-296-3p	1.78	MIMAT0004576	GAGGGUUGGGUGGAGGCUCUCC
miR-322-5p	1.64	MIMAT0000548	CAGCAGCAAUUCAUGUUUUGGA
miR-326-3p	2.04	MIMAT0000559	CCUCUGGGCCCUUCCUCCAGU
miR-330-3p	2.35	MIMAT0000569	GCAAAGCACAGGGCCUGCAGAGA
miR-330-5p	1.62	MIMAT0004642	UCUCUGGGCCUGUGUCUUAGGC
miR-342-3p	1.50	MIMAT0000590	UCUCACACAGAAAUCGCACCCGU
miR-375-3p	1.51	MIMAT0000739	UUUGUUCGUUCGGCUCGCGUGA
miR-376c-3p	1.86	MIMAT0003183	AACAUAGAGGAAAUUUCACGU
miR-378a-3p	1.54	MIMAT0003151	ACUGGACUUGGAGUCAGAAGG
miR-383-5p	1.66	MIMAT0000748	AGAUCAGAAGGUGACUGUGGCU
miR-410-3p	1.51	MIMAT0001091	AAUAUAACACAGAUGGCCUGU
miR-421-3p	1.57	MIMAT0004869	AUCAACAGACAUUAAUUGGGCGC
miR-455-3p	2.71	MIMAT0003742	GCAGUCCACGGGCAUAUACAC
miR-455-5p	1.53	MIMAT0003485	UAUGUGCCUUUGGACUACAUCG
miR-532-3p	3.81	MIMAT0004781	CCUCCCACACCCAAGGCUUGCA
miR-532-5p	1.62	MIMAT0002889	CAUGCCUUGAGUGUAGGACCGU
miR-1224-5p	3.40	MIMAT0005460	GUGAGGACUGGGGAGGUGGAG

**Table 2:** miRNAs putatively targeting *IRS-1*.

**miRNAs**	**Fold change**	**Accession**	**Sequence**
miR-15b-5p	1.62	MIMAT0000124	UAGCAGCACAUCAUGGUUUACA
miR-28a-3p	1.86	MIMAT0004661	CACUAGAUUGUGAGCUGCUGGA
miR-92b-3p	2.81	MIMAT0004899	UAUUGCACUCGUCCCGGCCUCC
miR-125a-5p	2.15	MIMAT0000135	UCCCUGAGACCCUUUAACCUGUGA
miR-149-5p	16.27	MIMAT0000159	UCUGGCUCCGUGUCUUCACUCCC
miR-151-5p	1.66	MIMAT0004536	UCGAGGAGCUCACAGUCUAGU
miR-155-5p	1.76	MIMAT0000165	UUAAUGCUAAUUGUGAUAGGGGU
miR-181a-5p	2.21	MIMAT0000210	AACAUUCAACGCUGUCGGUGAGU
miR-181b-5p	2.96	MIMAT0000673	AACAUUCAUUGCUGUCGGUGGGU
miR-181d-5p	1.52	MIMAT0004324	AACAUUCAUUGUUGUCGGUGGGU
miR-183-5p	1.51	MIMAT0000212	UAUGGCACUGGUAGAAUUCACU
miR-200c-3p	1.51	MIMAT0000657	UAAUACUGCCGGGUAAUGAUGGA
miR-296-3p	1.78	MIMAT0004576	GAGGGUUGGGUGGAGGCUCUCC
miR-322-5p	1.64	MIMAT0000548	CAGCAGCAAUUCAUGUUUUGGA
miR-325-3p	1.55	MIMAT0004640	UUUAUUGAGCACCUCCUAUCAA
miR-328-3p	2.20	MIMAT0000565	CUGGCCCUCUCUGCCCUUCCGU
miR-330-3p	2.35	MIMAT0000569	GCAAAGCACAGGGCCUGCAGAGA
miR-339-5p	2.13	MIMAT0000584	UCCCUGUCCUCCAGGAGCUCACG
miR-340-5p	1.54	MIMAT0004651	UUAUAAAGCAAUGAGACUGAUU
miR-342-3p	1.50	MIMAT0000590	UCUCACACAGAAAUCGCACCCGU
miR-370-3p	1.51	MIMAT0001095	GCCUGCUGGGGUGGAACCUGGU
miR-375-3p	1.51	MIMAT0000739	UUUGUUCGUUCGGCUCGCGUGA
miR-376c-3p	1.86	MIMAT0003183	AACAUAGAGGAAAUUUCACGU
miR-383-5p	1.66	MIMAT0000748	AGAUCAGAAGGUGACUGUGGCU
miR-410-3p	1.51	MIMAT0001091	AAUAUAACACAGAUGGCCUGU
miR-421-3p	1.57	MIMAT0004869	AUCAACAGACAUUAAUUGGGCGC
miR-423-3p	1.86	MIMAT0003454	AGCUCGGUCUGAGGCCCCUCAGU
miR-455-3p	2.71	MIMAT0003742	GCAGUCCACGGGCAUAUACAC
miR-455-5p	1.53	MIMAT0003485	UAUGUGCCUUUGGACUACAUCG
miR-466c-5p	2.09	MIMAT0004877	UGAUGUGUGUGUGCAUGUACAUAU
miR-466i-5p	1.78	MIMAT0017325	UGUGUGUGUGUGUGUGUGUG
miR-466m-5p	1.78	MIMAT0014882	UGUGUGCAUGUGCAUGUGUGUAU
miR-500-3p	2.29	MIMAT0003507	AAUGCACCUGGGCAAGGGUUCA
miR-501-3p	1.73	MIMAT0003509	AAUGCACCCGGGCAAGGAUUUG
miR-532-5p	1.62	MIMAT0002889	CAUGCCUUGAGUGUAGGACCGU
miR-669a-5p	2.31	MIMAT0003477	AGUUGUGUGUGCAUGUUCAUGUCU
miR-669f-5p	1.87	MIMAT0017327	AGUUGUGUGUGCAUGUGCAUGUGU
miR-669k-5p	1.85	MIMAT0017323	UGUGCAUGUGUGUAUAGUUGUGUGC
miR-669l-5p	1.87	MIMAT0009418	AGUUGUGUGUGCAUGUAUAUGU
miR-669m-5p	1.78	MIMAT0017346	UGUGUGCAUGUGCAUGUGUGUAU
miR-669p-5p	2.31	MIMAT0014889	AGUUGUGUGUGCAUGUUCAUGUCU
miR-1197-3p	1.51	MIMAT0005858	UAGGACACAUGGUCUACUUCU
miR-3102-5p	1.79	MIMAT0014933	GUGAGUGGCCAGGGUGGGGCUG
miR-3102-5p.2-5p	1.88	MIMAT0014934	GGUGGUGCAGGCAGGAGAGCC
miR-3473a	1.77	MIMAT0015645	UGGAGAGAUGGCUCAGCA
miR-3473b	1.71	MIMAT0020367	GGGCUGGAGAGAUGGCUCAG
miR-3473d	2.35	MIMAT0020632	CCACUGAGCCACUUUCCAGCCCUU

## Appendix A Supplementary material

Supplementary File 1 HFD - upregulated miRNAs (Excel file: HFD - upregulated miRNAs.xlsx).

.

Supplementary File 2 HFD - downregulated miRNAs (Excel file: HFD - downregulated miRNAs.xlsx).
